# Predictive Value of Neutrophil/Lymphocyte Ratio for Efficacy of Preoperative Chemotherapy in Triple-Negative Breast Cancer

**DOI:** 10.1245/s10434-015-4934-0

**Published:** 2015-10-28

**Authors:** Yuka Asano, Shinichiro Kashiwagi, Naoyoshi Onoda, Satoru Noda, Hidemi Kawajiri, Tsutomu Takashima, Masahiko Ohsawa, Seiichi Kitagawa, Kosei Hirakawa

**Affiliations:** Department of Surgical Oncology, Osaka City University Graduate School of Medicine, Osaka, Japan; Department of Diagnostic Pathology, Osaka City University Graduate School of Medicine, Osaka, Japan; Department of Physiology, Osaka City University Graduate School of Medicine, Osaka, Japan

## Abstract

**Background:**

The neutrophil/lymphocyte ratio (NLR) has been reportedly associated with prognosis in cancer patients by influencing both cancer progression and chemosensitivity. However, the correlation between NLR and the outcome of neoadjuvant chemotherapy (NAC) in breast cancer patients remains unclear.

**Methods:**

NLR was evaluated in 177 patients with breast cancer treated with NAC with 5-fluorouracil, epirubicin, and cyclophosphamide, followed by weekly paclitaxel and subsequent curative surgery. The correlation between NLR and prognosis, including the efficacy of NAC, was evaluated retrospectively.

**Results:**

NLR ranged from 0.5 to 10.6. Fifty-eight patients with low NLR (<3.0) had a higher pathological complete response (pCR) rate (*p* < 0.001) and were more frequently diagnosed with ER-negative/progesterone receptor (PR)-negative/HER2-negative (triple-negative) breast cancer (TNBC; *p* < 0.001) compared with patients with high NLR (≥3.0). Among TNBC patients who achieved pCR, disease-free survival (*p* = 0.006) and overall survival (*p* < 0.001) were significantly longer in patients with low NLR than in those with high NLR. Low NLR was associated with a significantly favorable prognosis in TNBC patients who achieved pCR, according to univariate analysis (*p* = 0.044, hazard ratio = 0.06).

**Conclusions:**

Low NLR may indicate high efficacy and favorable outcome after NAC in patients with TNBC.

**Electronic supplementary material:**

The online version of this article (doi:10.1245/s10434-015-4934-0) contains supplementary material, which is available to authorized users.

Treatment with neoadjuvant chemotherapy (NAC) increases the rate of breast-conserving surgery and reduces the risk of postoperative recurrence in patients with resectable breast cancer.^1^–^4^ Efficacy is enhanced by the combined use of anthracyclines and taxanes,^1^,^5^,^6^ and further improvements in therapeutic effects may be expected with the advent of molecular-targeting drugs.^7^,^8^ In addition, evaluation of the therapeutic effects of NAC can provide valuable information.

Breast cancer can be classified into the following phenotypes: estrogen receptor (ER)-positive (luminal, normal breast-like); human epidermal growth factor receptor (HER) 2-overexpressing; and basal-like, based on cDNA microarray analysis.^9^–^11^ The basal-like subtype displays characteristics of myoepithelial cells/basal cells, and often corresponds to so-called ER-negative/progesterone receptor (PR)-negative/HER2-negative (triple-negative) breast cancer (TNBC). Patients with TNBC do not benefit from endocrine or anti-HER2 therapy, and chemotherapy is the only treatment option.^12^–^16^ However, the efficacy of chemotherapy varies among patients with TNBC, and accurate methods for predicting the outcome not only help to identify the direct treatment effects, but also help to reduce adverse events caused by inappropriate treatment.

Abnormalities in white blood cell subpopulations, such as neutrophilia and lymphopenia, have been reported in tumor-bearing patients, and the neutrophil/lymphocyte ratio (NLR) has been found to be associated with prognosis in patients following radical surgery.^17^–^21^ Neutrophils are known to promote tumor cell proliferation, angiogenesis, and distant metastasis, while lymphocytes play a central role in the immune reaction against tumors. NLR may thus be related to both prognosis and chemosensitivity.^22^,^23^

This single-center, retrospective study aimed to evaluate NLR as a possible markers for predicting the outcome of NAC in patients with TNBC in a consecutive patient series treated with a standardized single protocol.

## Methods

### Patient Background

A total of 177 patients with resectable, early-stage breast cancer diagnosed as stage IIA (T1, N1, M0 or T2, N0, M0), IIB (T2, N1, M0 or T3, N0, M0), or IIIA (T1-2, N2, M0 or T3, N1-2, M0) were treated with NAC between 2007 and 2013. Tumor stage and T and N factors were stratified based on the TNM Classification of Malignant Tumors, UICC Sixth Edition.^24^ Breast cancer was confirmed histologically by core needle biopsy and staged by systemic imaging studies using computed tomography (CT), ultrasonography (US), and bone scintigraphy. Breast cancer was classified into subtypes according to the immunohistochemical expression of ER, PR, HER2, and Ki67. The cutoffs for ER positivity and PR positivity were both >0 % positive tumor cells with nuclear staining. Tumors with 3+ HER2 on immunohistochemical staining were considered to show HER2 overexpression; tumors with 2+ HER2 were further analyzed by fluorescence in situ hybridization; and those with HER2/CER17 ≥ 2.0 were also considered to exhibit HER2 overexpression. A Ki67-labeling index ≥ 14 % tumor cells with nuclear staining was determined to be positive.

All patients received a standardized protocol of NAC consisting of four courses of FEC100 (500 mg/m^2^ fluorouracil, 100 mg/m^2^ epirubicin, and 500 mg/m^2^ cyclophosphamide) every 3 weeks, followed by 12 courses of 80 mg/m^2^ paclitaxel administered weekly.^25^,^26^ Forty-five patients had HER2-positive breast cancer and were additionally administered weekly (2 mg/kg) or tri-weekly (6 mg/kg) trastuzumab during paclitaxel treatment.^27^ All patients underwent chemotherapy as outpatients. Therapeutic anti-tumor effects were assessed according to the Response Evaluation Criteria in Solid Tumors (RECIST) criteria.^28^ Pathological complete response (pCR) was defined as the complete disappearance of the invasive compartment of the lesion with or without intraductal components, including in the lymph nodes. Patients underwent mastectomy or breast-conserving surgery after NAC. All patients who underwent breast-conserving surgery were administered postoperative radiotherapy to the remnant breast. Overall survival (OS) time was the period from the initiation of NAC to the time of death from any cause. Disease-free survival (DFS) was defined as freedom from all local, loco-regional, and distant recurrences. All patients were followed up by physical examination every 3 months, US every 6 months, and CT and bone scintigraphy annually. The median follow-up period for the assessment of OS was 3.4 years (range, 0.6–6.0 years) and for DFS was 3.1 years (range, 0.1–6.0 years). The study protocol was approved by the Ethics Committee of Osaka City University (#926). Written informed consent was obtained from all subjects.

### Blood Sample Analysis

Peripheral blood was obtained at the time of diagnosis, before NAC. The numbers of white blood cells were determined using a hemocytometer. The percentages of different types of cells were determined using a Coulter LH 750 Hematology Analyzer (Beckman Coulter, Brea, CA, USA). NLR was calculated from the preoperative blood sample by dividing the absolute neutrophil count by the absolute lymphocyte count. On the basis of previous studies, an NLR value of 3.0 was used as the cutoff value to discriminate between high-NLR (≥3.0) and low-NLR (<3.0).^18^,^29^–^31^

### Statistical Analysis

Statistical analysis was performed using the SPSS version 19.0 statistical software package (IBM, Armonk, NY, USA). We examined the associations between NLR and clinicopathologic variables using χ^2^ tests. Multivariate analysis of pCR was carried out using a binary logistic regression model. The Kaplan–Meier method was used to estimate DFS and OS, and the results between groups were compared using log-rank tests. Multivariate analysis of prognostic factors was carried out using a Cox regression model. A *p* value <0.05 was considered significant. Cutoff values for different biomarkers included in this study were chosen before statistical analysis.^18^,^29^–^32^

## Results

### Clinicopathological Responses of Primary Breast Cancers to NAC

Clinical responses (pCR+ partial response) were observed in 151 patients (85.4 %). NAC-related pCR was observed in 67 patients (37.9 %). The pCR rates were 45.9 % (28/61) and 33.6 % (39/116) in patients with TNBC and with non-TNBC, respectively (Supplemental Table 1).

Among all cases, patients with pCR tended to have more favorable DFS (*p* = 0.254, log-rank) and OS (*p* = 0.221, log-rank) compared with those with non-pCR, though the differences were not significant (Supplemental Fig. 1A, B). However, TNBC patients with pCR had significantly better DFS (*p* = 0.043, log-rank) and OS (*p* = 0.049, log-rank) than those with non-pCR (Supplemental Fig. 1C, D). There was no significant difference in DFS (*p* = 0.964, log-rank) or OS (*p* = 0.975, log-rank) in relation to pCR among patients with non-TNBC (Supplemental Fig. 1E, F).

### Associations Between Clinicopathological Parameters and NLR

NLR was determined in every sample and ranged from 0.5 to 10.6 (mean, 2.3; median, 2.0; standard deviation 0.5). Fifty-eight patients were judged as having low NLR (32.8 %) and 119 as high NLR (67.2 %). Low NLR was significantly correlated with younger age (*p* = 0.038), premenopausal status (*p* = 0.037), pCR result (*p* < 0.001), and TNBC phenotype (*p* < 0.001) (Table 1). Clinicopathological features were further investigated in TNBC patients. TNBC patients with low NLRs had high Ki67 indexes (*p* = 0.002) and were significantly more likely to achieve pCR (*p* < 0.001). There was no significant correlation between NLR and any other tested clinicopathological parameters among TNBC patients (Table 2).

**Table 1:** Correlation between clinicopathological features and neutrophil to lymphocyte ratio in 177 all breast cancers

Parameters	NLR (*n* = 177)		(*p* value)
	High (*n* = 119)	Low (*n* = 58)	
Age at operation			0.038
≤56	52 (43.7 %)	35 (60.3 %)	
>56	67 (56.3 %)	23 (39.7 %)	
Menopause			0.037
Negative	42 (35.3 %)	30 (51.7 %)	
Positive	77 (64.7 %)	28 (48.3 %)	
Tumor size			0.053
≤2 cm	20 (16.8 %)	4 (6.9 %)	
>2 cm	99 (83.2 %)	54 (93.1 %)	
Lymph node status			0.552
Negative	26 (21.8 %)	15 (25.9 %)	
Positive	93 (78.2 %)	43 (74.1 %)	
Nuclear grade			0.469
1, 2	94 (79.0 %)	43 (74.1 %)	
3	25 (21.0 %)	15 (25.9 %)	
Ki67			0.292
≤14 %	53 (44.5 %)	21 (36.2 %)	
>14 %	66 (55.5 %)	37 (63.8 %)	
Intrinsic subtype			<0.001
TNBC	25 (21.0 %)	36 (62.1 %)	
non-TNBC	94 (79.0 %)	22 (37.9 %)	
Pathological			<0.001
pCR	34 (28.6 %)	33 (56.9 %)	
non-pCR	85 (71.4 %)	25 (43.1 %)	

*NLR* neutrophil-to-lymphocyte ratio, *TNBC* triple-negative breast cancers, *pCR* pathological complete response

**Table 2:** Correlations between neutrophil to lymphocyte ratio and clinicopathological parameters in 61 triple-negative breast cancers

Parameters	TNBC (*n* = 61)		(*p* value)
	High (*n* = 25)	Low (*n* = 36)	
Age at operation			0.069
≤56	8 (32.0 %)	20 (55.6 %)	
>56	17 (68.0 %)	16 (44.4 %)	
Menopause			0.274
Negative	7 (28.0 %)	15 (41.7 %)	
Positive	18 (72.0 %)	21 (58.3 %)	
Tumor size			0.610
≤2 cm	3 (12.0 %)	4 (11.1 %)	
>2 cm	22 (88.0 %)	32 (88.9 %)	
Lymph node status			0.084
Negative	2 (8.0 %)	9 (25.0 %)	
Positive	23 (92.0 %)	27 (75.0 %)	
Nuclear grade			0.198
1, 2	20 (80.0 %)	24 (66.7 %)	
3	5 (20.0 %)	12 (33.3 %)	
Ki67			0.002
≤14 %	13 (52.0 %)	5 (13.9 %)	
>14 %	12 (48.0 %)	31 (86.1 %)	
Pathological response			<0.001
pCR	2 (8.0 %)	26 (72.2 %)	
non-pCR	23 (92.0 %)	10 (27.8 %)	

*NLR* neutrophil-to-lymphocyte ratio, *TNBC* triple-negative breast cancers, *pCR* pathological complete response

### Correlation Between NLR and Prognosis After NAC

**Fig. 1:**
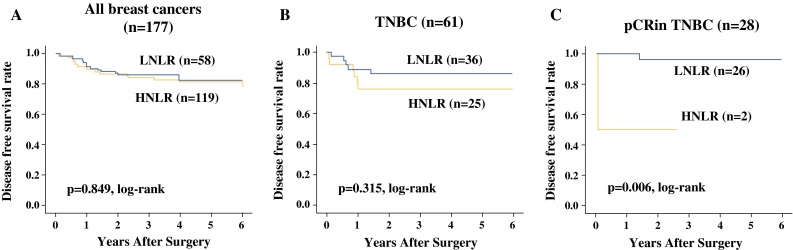
There was no significant difference in disease-free survival (DFS) in relation to NLR among all 177 breast cancer patients (**a**), or among 61 TNBC patients (**b**). However, among TNBC patients who achieved pCR, DFS (*p* = 0.006) was significantly longer in patients with low NLR after NAC, compared with patients with high NLR (**c**)

**Fig. 2:**
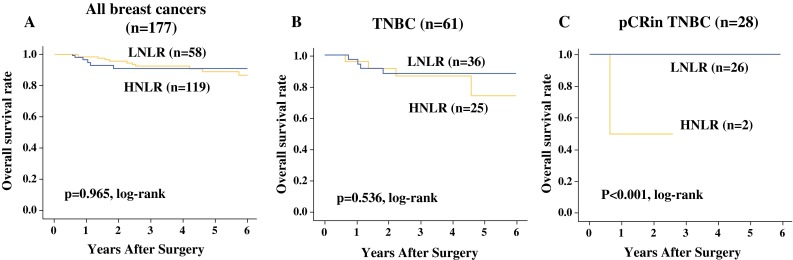
There was no significant difference in overall survival (OS) in relation to NLR among all 177 breast cancer patients (**a**), or among 61 TNBC patients (**b**). However, among TNBC patients who achieved pCR, OS (*p* < 0.001) was significantly longer in patients with low NLR after NAC compared with patients with high NLR (**c**)

There was no significant difference in DFS or OS among all 177 patients (Figs. 1a, 2a) or among the 61 TNBC patients (Figs. 1b, 2b) stratified by NLR. However, among TNBC patients who achieved pCR, DFS (*p* = 0.006, log-rank) and OS (*p* < 0.001, log-rank) were both significantly longer in patients with low NLR compared with patients with high NLR (Figs. 1c, 2c). There was no significant difference in DFS or OS in patients with non-pCR in relation to NLR (Supplemental Fig. 2A–F). On univariate analysis for recurrence, low NLR showed more favorable prognosis than high NLR (*p* = 0.044, hazard ratio (HR) = 0.06) (Table 3). However, multivariate analysis also demonstrated that low NLR status was not an independent factor to indicate significantly more favorable prognosis of the patients compared with high-NLR status (*p* = 0.173, HR = 0.09).

**Table 3:** Univariable- and multivariable analysis with respect to disease-free survival in 61 triple-negative breast cancers

Parameter	Univariable analysis			Multivariable analysis		
	Hazard ratio	95 % c.i.	*p* value	Hazard ratio	95 % c.i.	*p* value
Lymph node status						
Negative versus positive	0.26	0.16–4.08	0.334	0.45	0.14–14.44	0.651
Ki67 (%)						
≤14 versus >14	0.74	0.22–2.53	0.630	0.83	0.22–3.09	0.781
NLR						
Low versus high	0.06	0.00–0.92	0.044	0.09	0.00–2.89	0.173

*NLR* platelet-to-lymphocyte ratio, *c.i*. confidence interval

## Discussion

NLR scores in breast cancer patients have been reported previously.^18^,^19^ However, the current study specifically enrolled patients who were eligible for NAC. Our results confirmed the correlation between high NLR and older age or post-menopausal status, as reported previously,^18^,^19^ suggesting that NLR may be influenced by the patient’s systemic condition. We also found that patients with non-TNBC subtype had significantly higher NLRs. In the present study, the proportion of patients with non-TNBC was smaller (116/177, 65 %) than in previous reports.^18^,^19^ In addition, the characteristics of the patients with non-TNBC were biased because of the exclusion of patients unsuitable for NAC, such as older patients and those with early-stage or disseminated disease. The current study population was therefore not suitable for investigating the correlation between NLR and tumor subtype.

Azab et al. studied 465 patients and demonstrated significantly poorer survival in those with high (highest quartile) NLR.^19^ Dircan et al. reported similar findings.^33^ Several other studies have also shown a correlation between high NLR and prognosis in breast cancer patients with selected features, such as luminal A phenotype.^18^ NLR is known to be particularly influenced in patients with advanced-stage disease,^17^,^18^ though patients with stage I or IV disease were eliminated from the current study. These strict inclusion criteria may explain the apparent lack of a clear correlation between NLR and prognosis.

The main factor influencing prognosis in our series was the efficacy of NAC, which was significantly correlated with NLR. Patients with a low NLR had a significantly higher pCR rate compared with patients with a high NLR. Moreover, the relationship between NLR and the efficacy of NAC differed between subtypes; NLR was significantly associated with NLR in patients with TNBC, but not in those with non-TNBC. The meaning of pCR is known to differ according to breast cancer subtype, and significant survival benefit only occurs in TNBC patients who achieve pCR.^34^ Effective biomarkers for predicting the efficacy of NAC in TNBC patient are therefore needed. In the present study, low NLR showed a close correlation with a favorable prognosis in patients with TNBC who achieved pCR. This observation may indicate the value of measuring NLR in TNBC patients who require NAC in order to predict the efficacy of the treatment.

In TNBC, the proliferation marker Ki67 has been suggested as the pCR predictive biomarker of NAC.^35^ And the TNBC patients in the present study showed a correlation between low NLR and the high Ki67 index group. Therefore, we thought that patients with a low NLR achieved a high pCR rate. However, in the factor analysis, only low NLR was useful as a favorable prognosis factor in NAC.

Breast cancer is not generally regarded as an immune-related disorder. However, tumor-infiltrating lymphocytes and the expression of immune markers such as PD1, PDL1, and CTLA4 have recently been found to correlate with pCR in TNBC.^36^–^41^ NLR is relatively low in tumor subtypes with high lymphocyte activity, such as TNBC.^42^–^44^ Chemotherapy further activates the immune response in patients with low NLR, thereby accelerating the anti-tumor effect.^17^–^19^,^21^,^45^

Recent studies have suggested that TNBC can be classified into seven subtypes, depending on the gene expression profile,^44^,^46^ and sensitivities to treatment are thought to differ among the different subtypes.^34^ BL-1 is a basal-like subtypes that demonstrates a high cell mass in culture along with high expression levels of cell cycle and DNA-injury-responsive genes, and which shows high chemosensitivity. In contrast, the BL-2 basal-like subtype and luminal androgen receptor subtype, which overexpresses androgen receptors, both have low pCR rates.^34^ The immunomodulatory subtype overexpresses genes related to immune reactions and has demonstrated higher sensitivity to chemotherapy and a more favorable prognosis compared with other subtypes.^47^ The results of the present study suggest that NLR may be correlated with TNBC subtypes and different outcomes after successful NAC.

In the present study, NLR was closely correlated with prognosis in TNBC patients after successful NAC, suggesting that low NLR may represent a useful surrogate marker in patients with TNBC. NLR can be measured easily without the need for any special equipment or invasive procedures. Further prospective studies are therefore warranted to confirm these preliminary results and to investigate the correlations between TNBC characteristics or subtypes and NLR.

## Electronic supplementary material

Supplementary material 1 (DOCX 18 kb)

Supplemental Figure 1. Among all breast cancer cases, patients with pCR tended to have more favorable DFS (p = 0.254) and OS (p = 0.221) compared with those with non-pCR (A, B), though the differences were not significant. TNBC patients with pCR had significantly better DFS (p = 0.043) and OS (p = 0.049) than non-pCR patients (C, D). Among non-TNBC patients, there was no difference in DFS (p = 0.964) or OS (p = 0.975) in relation to pCR (E, F);Supplemental Figure 2. In patients with non-pCR, no significant survival periods were observed according to the difference in NLR (A-F). (PPTX 195 kb)
