# Supplementary material for: Predictive Value of Neutrophil/Lymphocyte Ratio for Efficacy of Preoperative Chemotherapy in Triple-Negative Breast Cancer
Source: Ann Surg Oncol. 2015 Oct 28;23:1104–10. doi: 10.1245/s10434-015-4934-0 (PMC4773470; doi:10.1245/s10434-015-4934-0)
Supplement: Supplementary file 1 — Supplementary material 1 (DOCX 18 kb) [file 10434_2015_4934_MOESM1_ESM.docx]

**Supplemental Table 1. Clinical response rate and pathological response rate to neoadjuvant chemotherapy.**

| pathological response | all breast cancer  (n=177) | TNBC  (n=61) | non-TNBC  (n=116) |
| --- | --- | --- | --- |
| pCR : pathological complete response |  |  |  |
| CR :complete response | 67 (37.9%) | 28 (45.9%) | 39 (33.6%) |
| non-pCR: non-pathological complete response |  |  |  |
| PR : partial response  SD : stable disease  PD : progressive disease | 84 (47.5%)  19 (10.7%)  7 (3.9%) | 21 (34.4%)  8 (13.1%)  4 (6.6%) | 63 (54.3%)  11 (9.5%)  3 (2.6%) |
| RR (CR+PR):response rate | 151 (85.4%) | 49 (80.3%) | 102 (87.9%) |

TNBC, triple-negative breast cancers.
