# Supplementary material for: Predictive Value of Neutrophil/Lymphocyte Ratio for Efficacy of Preoperative Chemotherapy in Triple-Negative Breast Cancer
Source: Ann Surg Oncol. 2015 Oct 28;23:1104–10. doi: 10.1245/s10434-015-4934-0 (PMC4773470; doi:10.1245/s10434-015-4934-0)
Supplement: Supplementary file 2 — Supplemental Figure 1. Among all breast cancer cases, patients with pCR tended to have more favorable DFS (p = 0.254) and OS (p = 0.221) compared with those with non-pCR (A, B), though the differences were not significant. TNBC patients with pCR had significantly better DFS (p = 0.043) and OS (p = 0.049) than non-pCR patients (C, D). Among non-TNBC patients, there was no difference in DFS (p = 0.964) or OS (p = 0.975) in relation to pCR (E, F);Supplemental Figure 2. In patients with non-pCR, no significant survival periods were observed according to the difference in NLR (A-F). (PPTX 195 kb) [file 10434_2015_4934_MOESM2_ESM.pptx]

## Slide 1
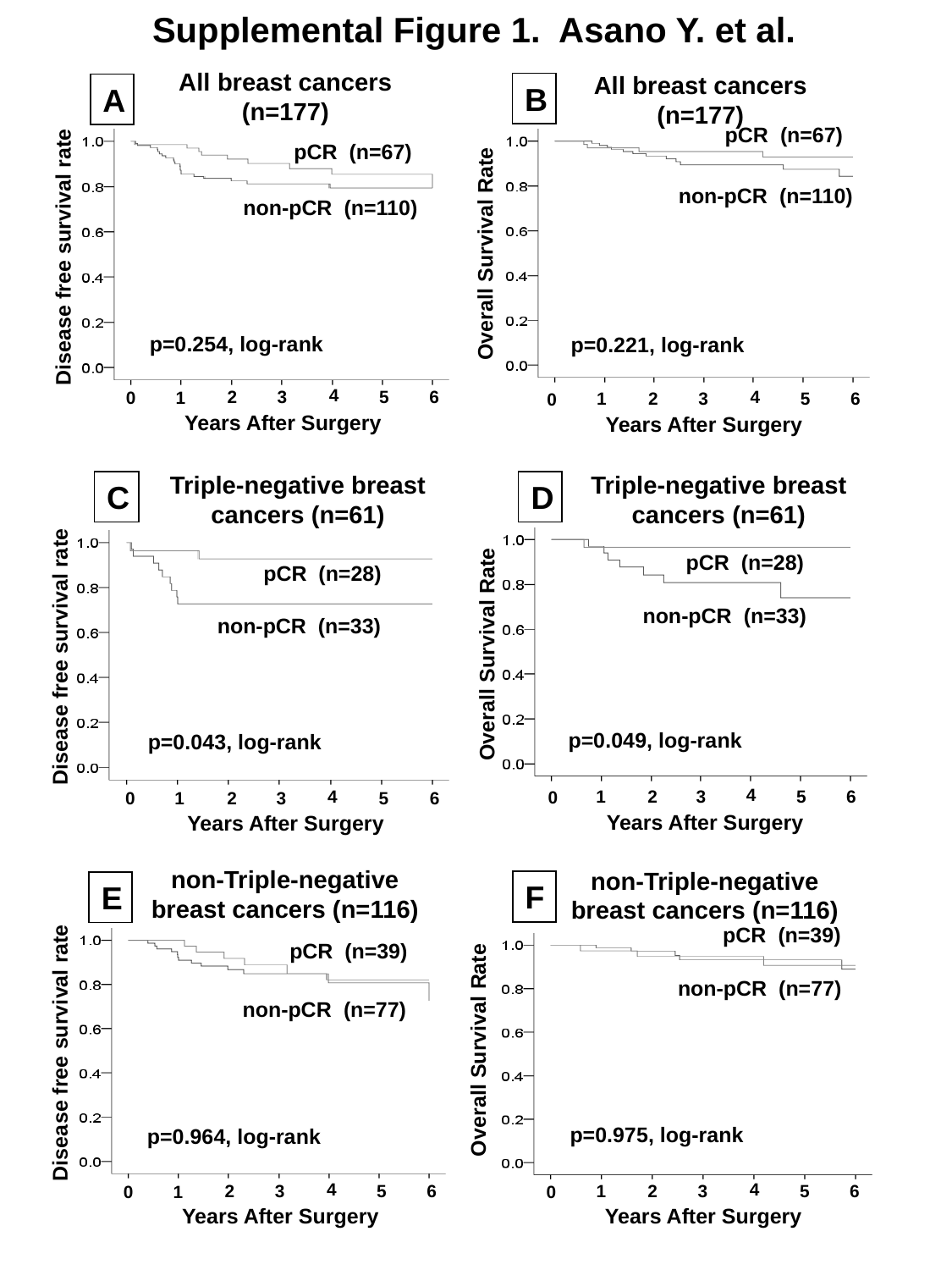

Supplemental Figure 1. Asano Y. et al.
All breast cancers (n=177)
All breast cancers (n=177)
B
A
pCR (n=67)
pCR (n=67)
non-pCR (n=110)
non-pCR (n=110)
Overall Survival Rate
Disease free survival rate
p=0.254, log-rank
p=0.221, log-rank
4
4
2
3
5
6
1
0
2
3
5
6
1
0
Years After Surgery
Years After Surgery
Triple-negative breast cancers (n=61)
Triple-negative breast cancers (n=61)
D
C
pCR (n=28)
pCR (n=28)
non-pCR (n=33)
non-pCR (n=33)
Overall Survival Rate
Disease free survival rate
p=0.049, log-rank
p=0.043, log-rank
4
4
2
3
5
6
1
0
2
3
5
6
1
0
Years After Surgery
Years After Surgery
non-Triple-negative breast cancers (n=116)
non-Triple-negative breast cancers (n=116)
F
E
pCR (n=39)
pCR (n=39)
non-pCR (n=77)
non-pCR (n=77)
Overall Survival Rate
Disease free survival rate
p=0.975, log-rank
p=0.964, log-rank
4
4
2
3
5
6
1
2
3
5
6
0
1
0
Years After Surgery
Years After Surgery

## Slide 2
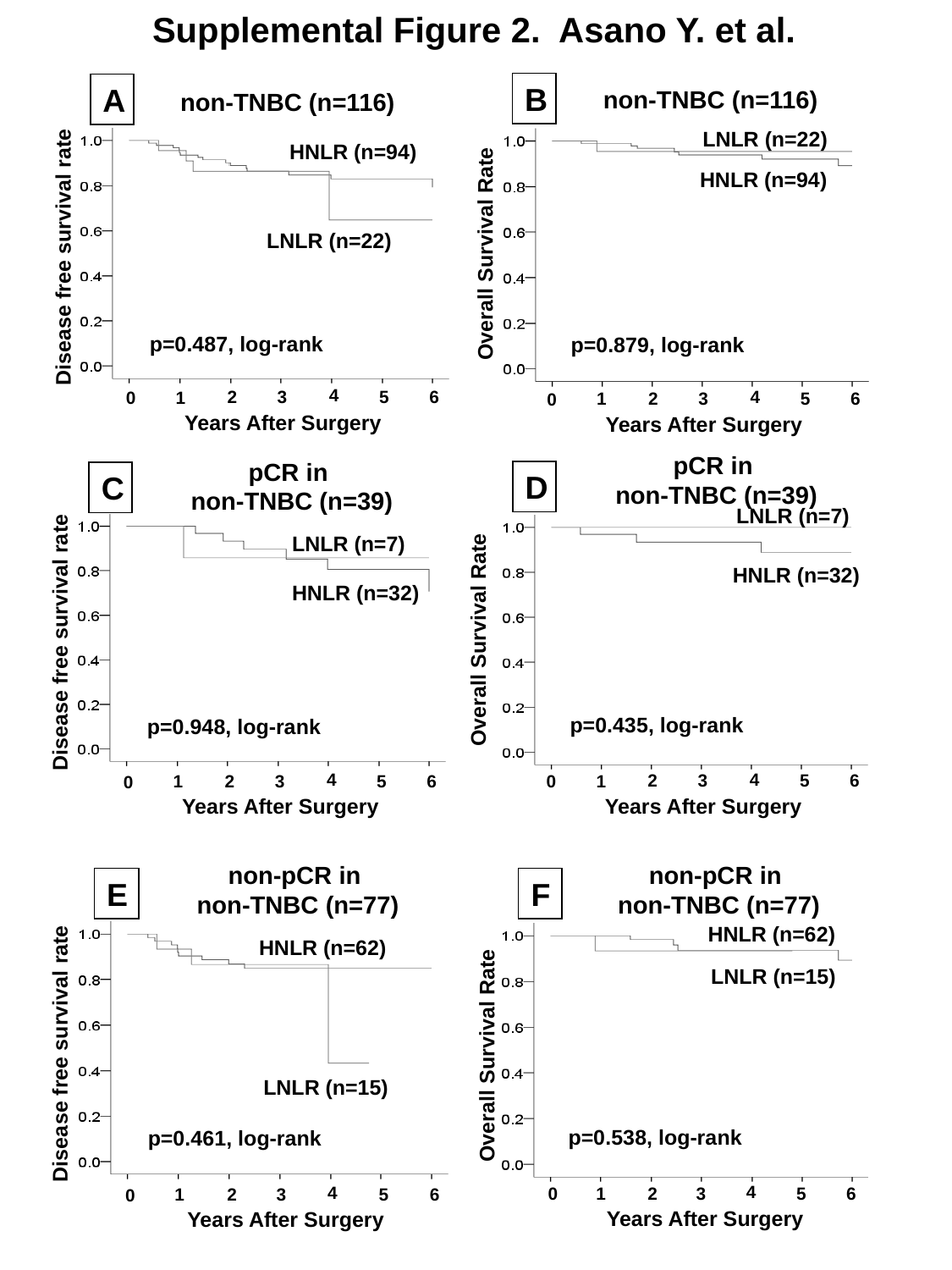

Supplemental Figure 2. Asano Y. et al.
B
non-TNBC (n=116)
LNLR (n=22)
HNLR (n=94)
Overall Survival Rate
p=0.879, log-rank
4
2
3
5
6
1
0
Years After Surgery
A
non-TNBC (n=116)
HNLR (n=94)
LNLR (n=22)
Disease free survival rate
p=0.487, log-rank
4
2
3
5
6
1
0
Years After Surgery
pCR in
non-TNBC (n=39)
D
HNLR (n=32)
Overall Survival Rate
p=0.435, log-rank
4
2
3
5
6
1
0
Years After Surgery
pCR in
non-TNBC (n=39)
C
LNLR (n=7)
HNLR (n=32)
Disease free survival rate
p=0.948, log-rank
4
2
3
5
6
1
0
Years After Surgery
LNLR (n=7)
non-pCR in
non-TNBC (n=77)
E
HNLR (n=62)
Disease free survival rate
LNLR (n=15)
p=0.461, log-rank
4
2
3
5
6
1
0
Years After Surgery
non-pCR in
non-TNBC (n=77)
F
HNLR (n=62)
LNLR (n=15)
Overall Survival Rate
p=0.538, log-rank
4
2
3
5
6
1
0
Years After Surgery
